# 3D printed hybrid scaffolds for bone regeneration using calcium methoxyethoxide as a calcium source

**DOI:** 10.3389/fbioe.2023.1224596

**Published:** 2023-08-17

**Authors:** Agathe Heyraud, Francesca Tallia, David Sory, Hung-Kai Ting, Anna Tchorzewska, Jingwen Liu, Hannah L. Pilsworth, Peter D. Lee, John V. Hanna, Sara M. Rankin, Julian R. Jones

**Affiliations:** ^1^ Department of Materials, Imperial College London, London, United Kingdom; ^2^ Faculty of Medicine, Imperial College London, National Heart and Lung Institute, London, United Kingdom; ^3^ Department of Mechanical Engineering, Faculty of Engineering Science, University College London, London, United Kingdom; ^4^ Department of Physics, University of Warwick, Coventry, United Kingdom

**Keywords:** sol-gel hybrid, bone regeneration, bioactive, Direct Ink Writing, calcium

## Abstract

**Introduction:** Hybrids consist of inorganic and organic co-networks that are indistinguishable above the nanoscale, which can lead to unprecedented combinations of properties, such as high toughness and controlled degradation.

**Methods:** We present 3D printed bioactive hybrid scaffolds for bone regeneration, produced by incorporating calcium into our “Bouncy Bioglass”, using calcium methoxyethoxide (CME) as the calcium precursor. SiO_2_-CaO_CME_/PTHF/PCL-diCOOH hybrid “inks” for additive manufacturing (Direct Ink Writing) were optimised for synergy of mechanical properties and open interconnected pore channels.

**Results and Discussion:** Adding calcium improved printability. Changing calcium content (5, 10, 20, 30, and 40 mol.%) of the SiO_2_-CaO_CME_/PTHF/PCL-diCOOH hybrids affected printability and mechanical properties of the lattice-like scaffolds. Hybrids containing 30 mol.% calcium in the inorganic network (70S30C_CME_-CL) printed with 500 µm channels and 100 µm strut size achieved the highest strength (0.90 ± 0.23 MPa) and modulus of toughness (0.22 ± 0.04 MPa). These values were higher than Ca-free SiO_2_/PTHF/PCL-diCOOH hybrids (0.36 ± 0.14 MPa strength and 0.06 ± 0.01 MPa toughness modulus). Over a period of 90 days of immersion in simulated body fluid (SBF), the 70S30C_CME_-CL hybrids also kept a stable strain to failure (~30 %) and formed hydroxycarbonate apatite within three days. The extracts released by the 70S30C_CME_-CL hybrids in growth medium did not cause cytotoxic effects on human bone marrow stromal cells over 24 h of culture.

## Introduction

Non-union bone defects occur in up to 10% of all fracture cases (Calori et al., 2011), and may form critical size defects which do not correctly heal if unaided (2–2.5 times the diameter of the affected bone) (Lasanianos et al., 2010). These often require reconstructive grafts to fill the defect and repair the bone. The gold standard is autografts; however, the procedure has limitations such as donor site morbidity, pain, and the requirement of a second operation site to harvest the bone, increasing recovery time and risks of infection (Baino and Vitale-Brovarone, 2011). The amount of bone that can be harvested is limited and has mismatched mechanical properties compared to long bones (Lasanianos et al., 2010; Jones, 2013), due to being non-load bearing and may poorly fit the defect site. Bone grafts are also not recommended when the defect exceeds 4–5 cm in length as partial resorption of the graft and revascularisation result in weakness and susceptibility to fractures (Lasanianos et al., 2010). Therefore, non-union bone defects are an unmet clinical need. Novel techniques have been used for long-bone diaphyseal defects (>5 cm), such as vascularised bone grafts (Lasanianos et al., 2010), or induced membrane technique with external fixators (Roddy et al., 2018; Masquelet et al., 2019). This however highlights the need for more specialised interventions. Synthetic biomaterial medical devices could meet this unmet need for scaffolds (temporary templates) and: share load with the host bone; bond to the host bone; stimulate osteoprogenitor cells to produce new bone; support vascularised bone ingrowth in a 3D structure; biodegrade as the bone remodels (Jones, 2013).

Recent medical devices for bone regeneration focus on biodegradable materials, with the aim of bone replacing the graft as the material degrades, followed by continued bone remodelling. Bioglass and silicon substituted hydroxyapatite have been proven to stimulate osteogenesis *in vitro* (Xynos et al., 2001) and improved bone regeneration *in vivo* (Oonishi et al., 2000; Midha et al., 2013) compared to other synthetic bone grafts, but while they have good compressive strengths, they are brittle (Jones et al., 2006a; Poologasundarampillai et al., 2016). A hypothesis is that choosing a scaffold material that can share cyclic loading with the host tissue can avoid stress shielding and promote high quality bone regeneration (Wei et al., 2020). Biodegradable polymers (natural: collagen, or synthetic: aliphatic polyesters), provide elasticity but not suitable strength (Wei et al., 2020). Combining organic polymers and inorganic bioactive ceramics or glasses as composites would be a strategy for increasing the toughness of bioactive ceramics or glasses (Koons et al., 2020). However, this leads to bioactive phases being hidden from the *in vivo* environment and heterogeneous degradation rates between the phases, resulting in poor mechanical properties as the scaffold degrades during the bone regeneration process (Jones, 2013). A hybrid differs from composites as their inorganic and organic components are indistinguishable above the nanoscale (Jones, 2013), making them single phase materials. The hypothesis is that the fine scale integration of the co-networks will enable the hybrid biomaterial to degrade as one phase and allow cells to interact with the organic and inorganic networks simultaneously, with no masking of the bioactive phase (Valliant and Jones, 2011). Hybrid biomaterials are synthesised by incorporating a polymer into the sol-gel process, which is usually based on the hydrolysis of silicate based alkoxides such as tetraethylothosilicate (TEOS), prior to the gelation of the sol, which forms a silicate network. The synthesis and subsequent drying are carried out below 60°C to prevent polymer burnout or thermal degradation (Tallia et al., 2018). The specification of the organic polymer for the hybrid is important, as it must be: soluble in the sol; tough; able to form dynamic and covalent bonds with the inorganic network; degradable at a controllable rate. For hybrids designed for use as biomaterials, they must have covalent bonds between the organic and inorganic networks, otherwise water will penetrate and force the chains apart, causing dissolution (Poologasundarampillai et al., 2010). Covalent bonding can be achieved through covalent coupling molecules, such as (3-glycidyloxypropyl) trimethoxysilane (GPTMS), (3-isocyanatopropyl) triethoxysilane (ICPTS), or by co-polymerisation techniques (Tallia et al., 2018). Early examples used ICPTS as the coupling agent for PCL containing class II hybrids (Tian et al., 1996; Tian et al., 1997; Tian et al., 1998; Rhee et al., 2002; Rhee, 2004), but mechanical properties were low. Others used gelatin as the polymer, with its carboxylic groups opening the epoxide ring of the coupling agent, GPTMS. The gelatin therefore presented siloxane groups to the silica sol (Mahony et al., 2010). Hybrids with caprolactone co-polymers, GPTMS, and silica were also synthesised and showed adhesion of pre-osteoblast cells to the material (Sang et al., 2018).

For the inorganic network of the hybrids to have the bioactive properties of bioactive glass, calcium must be incorporated into the silicate network (Tallia et al., 2022). This will trigger hydroxycarbonate apatite (HCA) layer formation for bone bonding and release of calcium ions for osteogenic stimulation of cells (Jones, 2013). However, the addition of calcium to the inorganic component of the hybrid poses a challenge: the common calcium source used for sol-gel glass production is calcium nitrate, as it is soluble in the sol. However, it requires a heat treatment higher than 400°C to burn off toxic nitrate by-products and incorporate the calcium into the silicate network (Lin et al., 2009; Jones, 2013). While calcium can be introduced into hybrids at low temperatures as non-toxic calcium salts, such as calcium chloride, the calcium does not bond into the network, making its release uncontrolled on immersion in fluids. Low temperature (<60°C) calcium incorporation in hybrids is needed to prevent the polymer component from degrading (Valliant and Jones, 2011). As an alternative to calcium salts, calcium alkoxides have been used, such as calcium methoxyethoxide (CME) and calcium ethoxide (CE). CME has been shown to incorporate into the silica network at temperatures below 60 °C, where calcium salts did not (Yu et al., 2012). It was hypothesised that the CME hydrolyses when added to the hydrolysed TEOS solution, incorporating calcium into the wet gel. As the silica cross-links during ageing and drying, it maintains calcium in its network (Yu et al., 2012). Using calcium alkoxide instead of nitrate as the precursor for sol-gel glass synthesis was shown to produce thicker and more homogeneous HCA surface layers on glasses (Rámila et al., 2002). CME was successfully incorporated into a SiO_2_-CaO/poly (γ-glutamic acid) bulk hybrids (Poologasundarampillai et al., 2014), SiO_2_-CaO/polyethylene glycol bulk hybrids (Li et al., 2015), and SiO_2_-CaO/PTHF/PCL-diCOOH bulk hybrids (Tallia et al., 2022) which all showed apatite formation after 3 days in simulated body fluid (SBF) and promising cell response. CE was also investigated as another calcium source in class II silica-gelatin hybrids with GPTMS as the coupling agent (Dieudonné et al., 2014; Lao et al., 2016). Calcium alkoxides being highly sensitive to water meant the choice of water-soluble gelatin limited the synthesis process to avoid fast gelation, leading to uneven distribution of the calcium (Dieudonné et al., 2014; Lao et al., 2016).

None of these calcium-containing hybrid compositions were optimised for additive manufacturing. Tallia et al. recently reported hybrids of the SiO_2_/PTHF/PCL-diCOOH composition that could be printed through Direct Ink Writing and showed excellent resistance to cyclic loads (Tallia et al., 2018). The size of the pore channels was found to be critical for the specific tissue engineering application. When scaffolds were printed with ∼250 µm wide pore channels, bone marrow stem cells cultured in the scaffolds progressed down the chondrogenic route, producing articular-like cartilage matrix rich in collagen Type II, Aggrecan, Sox9, and glycosaminoglycans, indicative of articular cartilage matrix. When the pore channels were increased to 500 μm, the cells produced matrix that was collagen Type I rich (Li et al., 2020). This underlined the importance of the geometry, as well as degradation by-products to trigger cell signalling and tissue regeneration. For bone regeneration applications, it was therefore important to consider the effect of calcium addition to the hybrid structure. Calcium was first successfully incorporated into the SiO_2_-CaO/PTHF/PCL-diCOOH hybrids using CME with a 60:40 TEOS:CME molar ratio (Tallia et al., 2022), but only in the form of cylindrical monoliths, reaching stress and strain to failure of 60 MPa and 55%, respectively (Tallia et al., 2022), compared to calcium free SiO_2_/PTHF/PCL-diCOOH hybrid monoliths with similar inorganic/organic ratio reaching only 3 MPa and 28%, respectively (Tallia et al., 2018). This increase in strength and toughness due to the addition of calcium showed a real potential for use in bone regeneration.

The aim of this work was to build on the previous research to develop a new hybrid with calcium fully incorporated into the silicate network and synthesise it into an “ink” for Direct Ink Writing to produce open channel interconnective scaffolds suitable for vascularised bone regeneration (>400 μm) with mechanical properties similar to porous bone. The scaffold should also trigger apatite formation *in vitro* and maintain mechanical properties during the biodegradation.

## Materials and methods

### Materials

All materials were obtained from Sigma Aldrich (Dorset, UK) and VWR UK, unless specified otherwise.

### Synthesis of calcium methoxyethoxide (CME)

CME was prepared following the process established by Pickup et al. (2009): 2 g of calcium was reacted with 48 mL of anhydrous 2-methoxyethanol at 80°C under Argon for 24 h; the solution was then centrifuged for 20 min at 6,000 rpm to remove unreacted calcium metal and deposit. A transparent dark red solution was obtained. To measure its concentration, 1 mL of solution was transferred into a platinum crucible and heated to 1,050°C for 10 h: the solvent evaporated, and the CME converted to CaO. The concentration was calculated as a ratio of the mass of CaO and the molecular weight of CaO. The CME concentration used in this work was 1 M.

### Synthesis of SiO_2_-CaO_CME_/PTHF/PCL-diCOOH hybrid inks for Direct Ink Writing

**FIGURE 1:**
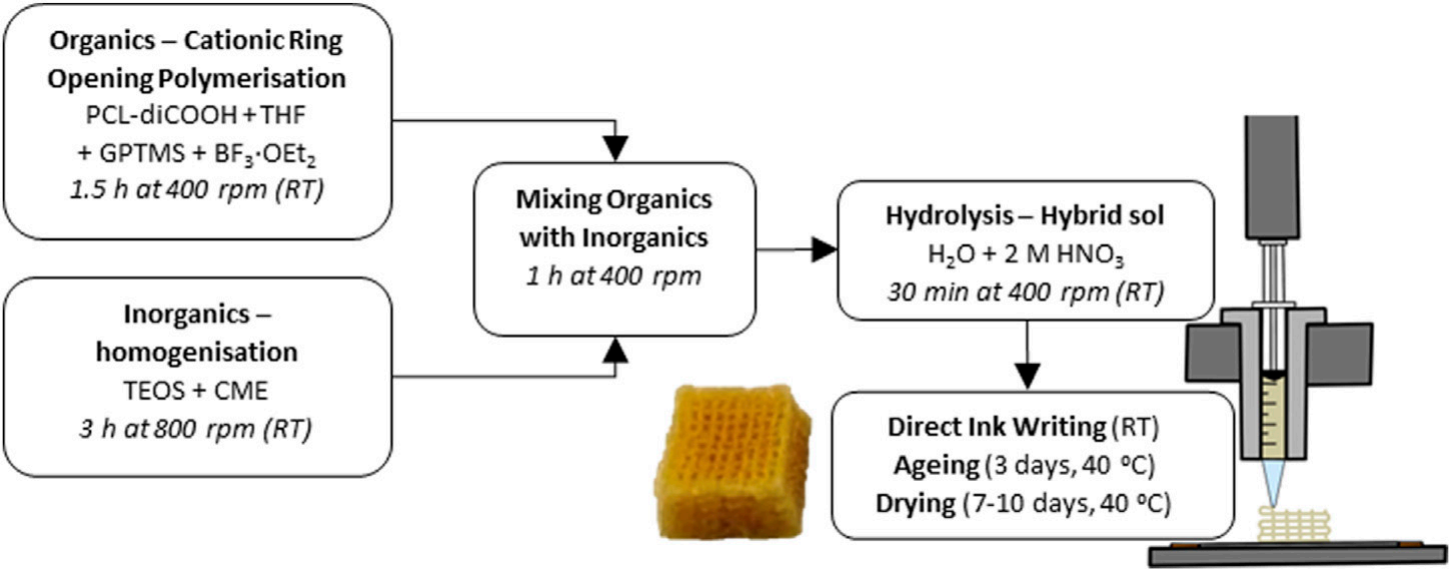
Flow chart of the complete SiO_2_-CaO_CME_/PTHF/PCL-diCOOH hybrid scaffold synthesis at room temperature (RT).

The synthesis of the hybrid ink was a two-step procedure developed previously (Tallia et al., 2018), starting with a TEMPO oxidation of the PCL diol to produce a dicarboxylic acid, PCL-diCOOH. The -COOH group was necessary to react with the coupling agent of the hybrid, GPTMS, to form covalent bonds between the silica network and the polymers (Tallia et al., 2018). PCL-diCOOH was used in the sol-gel hybrid synthesis in the organic precursor solution, Figure 1, consisting of PCL-diCOOH (1 mol), (3-glycidyloxypropyl) trimethoxysilane (GPTMS, 2 mol) and boron trifluoride diethyletherate (BF_3_·EOt_2_, 0.5 mol) in tetrahydrofuran (THF, 100 mg mL^-1^ with respect to PCL-diCOOH). This solution was stirred at room temperature for 1.5 h to allow the polymerisation of THF to occur, forming PTHF. GPTMS acted as the initiator for *in situ* cationic ring-opening polymerisation of the THF solvent. In parallel, the inorganic solution was prepared, with TEOS as a silica precursor, using a TEOS:PCL ratio of 70:30 wt.% to achieve a final inorganic:organic (I:O) ratio of 20:80 wt.%. The TEOS was mixed with the CME solution, a TEOS:CME ratio of 60:40 mol.% was initially used but altered throughout the work, down to 95:5 mol.%. The TEOS and CME were mixed at room temperature for 3 h. The organic solution was added dropwise to the inorganic sol and left to stir at room temperature (RT) for a further hour at 400 rpm^24^. After which, the deionised water was added in stoichiometric volume to hydrolyse the TEOS and GPTMS, followed by 2 M nitric acid (1/3 volume of water). The sol was stirred for 30 min before removing the lid to increase the evaporation of excess solvent, this part was found to take from 10 up to 45 min, varying with temperature and humidity. When the appropriate viscosity was achieved it was then poured into 3 mL Luer-lock plastic syringes and stored in a freezer at −82°C.

### Direct Ink Writing of SiO_2_-CaO_CME_/PTHF/PCL-diCOOH hybrid

The SiO_2_-CaO_CME_/PTHF/PCL-diCOOH sol-gel ink syringes were defrosted. Once the correct viscosity (degree of gelation) was reached for printing (∼4 h), the syringe was placed in the Direct Ink Writing machine (Robocaster, 3d Inks LLC, USA), connected to a computer with the RoboCAD software (3d Inks LLC, USA). The strut size was determined by the nozzle used, and the strut separation and layer thickness by the scaffold design. The ideal viscosity, which was visually assessed, was reached when the ink was liquid enough to flow through the nozzle but viscous enough to hold its shape without collapsing. This ongoing gelation was utilised to form bonds between the printed layers. The printing window (length of time for which the ink could be printed) was between 1 and 2 h, increasing as calcium content increased. The printing speed and deposition rate also helped to determine the final strut size; with faster printing speed the ink filament could break. A speed of 10 mm s^-1^ and deposition rate of 0.05 mL min^-1^ were used. The latter was held constant during printing by an automatic force adjustment applied to the plunger on the *z*-axis, as the ink viscosity and resistance to extrusion increased with gelation. Strut spacing of 1 mm guaranteed a final pore size of 400–500 μm, post drying. The layer thickness, or z-spacing, was set at 0.20 mm, matching the tip size. The printing substrate needed to allow for stability of the scaffold during extrusion but facilitate the removal after printing. Greaseproof paper was taped on a flat metal plate and used as the printing substrate. Once the scaffolds were printed, they were carefully removed from the substrate and placed in poly (methyl pentene), PMP, airtight pots for the ageing and drying process at 40°C. The ageing process lasted 3 days in an airtight container, followed by gradual loosening of the lid by a quarter of a revolution every day for 7 days, or until fully opened, to slowly dry the scaffolds.

### Characterisation of the hybrid scaffolds

To evaluate the architecture of the hybrid scaffolds, Scanning Electron Microscopy (SEM, JEOL 6010 LA) was used in secondary electron mode with a voltage of 20 kV, a working distance between 13 and 20 mm and spot size between 40 and 60 μm. The samples were fixed on aluminium holders using carbon tape and coated in a 10—15 nm layer of gold using a EMITECH K575X Peltier cooled coater. The top surface and cross sections were imaged after manually cutting with a sharp blade to investigate the horizontal and vertical pore channels of the scaffolds. Post simulated body fluid (SBF) immersion, SEM was used to analyse signs of apatite formation or surface degradation.

To characterise the porosity percentage and interconnectivity of channels, 60S40C_CME_-CL scaffolds (Table 1) were scanned using X-ray microtomography (µCT, Nikon XTH225 ST) at 70 kV and 140 μA, with a voxel size of 4.0 µm. The µCT images were reconstructed (Nikon’s CT Pro 3D) and a sub volume of interest (VOI, 2,600 × 2,600 × 2,600 μm) was defined for quantification. First a 3 × 3 × 3 median filter was applied to reduce noise (Yue et al., 2011), then manual thresholding was performed, selecting the mid-point between attenuation peaks using Avizo (version 2021). “Label analysis” in Avizo was utilised for the measurement of porosity and interconnectivity (percentage of pores connected with each other and the exterior). Pore sizes and the strut equivalent diameter of 60S40C_CME_-CL scaffolds were measured using the open-source image processing program ImageJ with the BoneJ plugin (Doube et al., 2010). Two types of thickness maps were made: one illustrating pore size and the other strut equivalent diameter (Atwood et al., 2004).

**TABLE 1:** SiO_2_-CaO_CME_/PTHF/PCL-diCOOH hybrids synthesised with varying compositions and final I:O ratios.

Hybrid name (xSyC_CME_-CL, where x:y is the TEOS:CME molar ratio)	TEOS:CME ratio (mol.%)	TEOS:PCL-diCOOH ratio (wt.%)	I:O ratio (wt.%)
60S40C_CME_-CL	60:40	70:30	23.1:76.9
70S30C_CME_-CL	70:30	72.5:27.5	25.5:74.5
80S20C_CME_-CL	80:20	75:25	23.8:76.2
90S10C_CME_-CL	90:10	77.5:22.5	26.3:73.7
95S5C_CME_-CL	95:5	78.75:21.25	27.2:72.8
100S-CL	100:0	80:20	25.1:74.9

Fourier Transform Infra-red (FTIR) spectroscopy was used to verify the presence of the functional groups corresponding to the organic and inorganic components of the hybrid system and investigate the variation in the hybrid chemical structure and bonding due to the different addition of calcium. This technique also identified compositional changes after immersion in SBF. A Thermo Scientific Nicolet iS10 FTIR equipped with Smart Golden Gate for Single-Reflection Diamond ATR Analysis, with OMNIC software was used. 64 scans were collected at a resolution of 4 cm^-1^ for a spectral range of 400—4,000 cm^-1^. Solid samples were usually prepared by manually grinding them into a fine powder.

Simultaneous Differential Scanning Calorimetry and Thermogravimetric Analysis (DSC/TGA) were performed on Netzsch Jupiter STA 449C instrument with *Proteus* software to process the acquired data. The hybrid samples were manually ground to a fine powder, and between 10 and 15 mg was placed in a platinum crucible, the reference being an empty platinum crucible. A heating rate of 10°C min^_1^ over a temperature range of 20–800°C under continuously flowing air was used. This technique was used to analyse the hybrids final I:O ratio and finding the characteristic burn-out temperature of both polymers present in the system: PCL-diCOOH and PTHF. The I:O ratios were also evaluated after various timepoints of immersion in SBF.

Compression testing was done to assess maximum stress and strain of the 3D printed hybrid scaffolds. A sharp blade was used to cut the scaffolds to 5 × 5 × 5 mm^3^ cubes, and the load was applied perpendicular to the plane of deposition during printing. A Bose Electroforce Series III mounted with a 450 N load cell was used for compression testing, with the Wintest software to collect the data. The displacement rate used was 0.5 mm min^-1^. The engineering and true stress and strain at yield were calculated. Cyclic loading was also performed on scaffolds of the same dimensions to show their ability to recover deformation in a specific strain range. The test comprised of 10 cycles, all in the same conditions, in a strain-controlled manner to compress each sample to 20% of their original measured height. The loading and unloading steps were both performed at 0.5 mm min^-1^, similar to the compression to failure tests. A dwell time of 30 s between each cycle was programmed to enable complete recovery of the deformation after loading. DMA (Dynamic Mechanical Analysis) was performed on the same scaffold dimensions with the Bose Electroforce Series III used in parallel with the Wintest DMA software. The tests were done at strain ranges of 1%–5%, 5%–9% and 9%–13%, individually calculated for each scaffold from their measured height, collecting data at frequencies of 0.1, 1 and 10 Hz. DMA was performed to analyse the viscoelastic behaviour of the scaffolds and compare the stiffness of hybrids with varying calcium content and after immersion in SBF at various timepoints.

X-ray Diffraction (XRD) measurements were performed using a Bruker D2 PHASER desktop diffractometer, the data was analysed with a PANalytical X’Pert HighScore software. A Cu Kα tube anode was used (*λ* = 1.5418 Å) and the generator settings fixed at a voltage of 30 kV and current intensity of 10 mA. A nickel filter was used to remove Kβ, though still visible from most intense peaks. Each pattern was recorded in the range of 5 to 120°, this is specified if changed to analyse a narrower range of angles. The step size and time per step were set at 0.035° and 0.35 s step^−1^. Minimal sample preparation is required, hybrid samples were usually manually ground to a fine powder and flattened in a single crystal silicon sample holder, to prevent it showing background noise. This method provided information on the presence of crystallised apatite on otherwise amorphous hybrid scaffolds after immersion in SBF.

A Thermo Scientific ICAP 6300 Inductively coupled plasma-optical emission spectroscopy (ICP-OES) with autosampler was used in parallel with the iTEVA software to determine the concentration of Ca, Si and P in SBF solution after immersion of hybrid scaffolds. The aim was to analyse potential apatite formation on the hybrids for bone regeneration, as well as monitor the slow release of silica and calcium ions due to the controlled degradation of the hybrid (Tallia et al., 2022). Due to the high content of calcium in SBF itself, the samples were diluted by a factor of 10 with DI water (1 mL of the aqueous sample and 9 mL of DI water). The standard solutions for calibration were prepared containing Si, Ca, and P at 0, 0.1, 0.2, 0.4, 0.8, 1, 5, 10 and 20 μg mL^-1^. For the dissolution studies, timepoints of 0–8 h, 1, 3, 7, 21, 30, 60 and 90 days were selected to analyse the effect of immersion of hybrid scaffolds with different calcium content in SBF. SBF was used, as opposed to phosphate buffered saline (PBS) or tris(hydroxymethyl)aminomethane (TRIS), as the deposition of apatite on the bone scaffold was investigated. Scaffolds were cut to the desired size (5 × 5 × 5 mm^3^) and weighed to measure mass loss post dissolution. They were then rinsed three times in DI water prior to the immersion in SBF to ensure no reaction by-product were left over (such as BF_3_·EOt_2_) and placed in SBF at a 1.5 mg mL^-1^ scaffold mass to SBF volume ratio. This concentration is commonly used for testing apatite formation on bioactive glasses in SBF (Jones et al., 2001; Tallia et al., 2022). The scaffolds were then kept in an incubator at 37 °C and 120 rpm (Maçon et al., 2015).

All ^29^Si Magic-Angle-Spinning Nuclear Magnetic Resonance (MAS NMR) measurements were performed at 7.05 T using a Bruker Avance III HD-300 spectrometer operating at a^29^Si Larmor frequency of 59.5 MHz. These experiments were undertaken using a Bruker 7 mm HX probe which enabled a MAS frequency of 5 kHz for all ^29^Si single pulse experiments performed to assess the Si speciation quantitatively. The pulse time calibration was performed on solid kaolinite (Al_2_O_3_·2SiO_2_·2H_2_O) from which a π/2 pulse time of 6.0 μs was measured. All ^29^Si MAS NMR data were acquired using a π/2 nutation angle, a recycle delay of 240 s, and a heteronuclear ^1^H/^29^Si decoupling field strength of 80 kHz during data acquisition. The reported ^29^Si chemical shifts were referenced against the IUPAC recommended primary reference of Me_4_Si (1% in CDCL_3_, δ_iso_ = 0.0 ppm), via a secondary solid kaolinite standard which has a known shift of δ_iso_ = −92.0 ppm (Harris et al., 2002). The degree of condensation of the silica network, Dc, Equation 1, was calculated from quantitative measurement of the Q^n^ structures derived from TEOS (Si(OSi)_n_ (OR)_4-n_ species, with R being Ca or H) and T^n^ structures derived from the coupling agent GPTMS (C-Si(OSi)_n_ (OR)_3-n_ species, with R being Ca or H), from the ^29^Si MAS NMR data (Connell et al., 2014).

$$D_{c}=\left(\left[\frac{{4Q}^{4}+{3Q}^{3}+{2Q}^{2}}{4}\right]+\left[\frac{{3T}^{3}+{2T}^{2}+T^{1}}{3}\right]\right)$$ (1)

### Cell culture study: Conditioned medium, cell seeding, and plate preparation for *in vitro* cultures

Discs of 8 mm diameter were punched out of 1 mm thick 70S30C_CME_-CL and 90S10C_CME_-CL hybrid monoliths. The discs were sterilised by 3 washing cycles in DI water, 70% and 100% ethanol, then irradiated under UV light for 3 h and air dried under sterile conditions for 24 h.

The 70S30C_CME_-CL and 90S10C_CME_-CL hybrid conditioned media and vehicle medium (VC, fresh serum-free Dulbecco’s Modified Eagle Medium**,** DMEM) were prepared as per ISO 10993–12 (ISO 10993-12:2021 Biological evaluation of medical devices—Part 12: Sample preparation and reference materials, 2021) (3 discs mL^-1^ of fresh serum-free DMEM to give 300 mm^2^ mL^-1^, 72 h incubation at 37 °C). The conditioned media were sterilised by filtration through a 0.2 μm non-pyrogenic sterile 28 mm syringe surfactant-free cellulose acetate filter (#431219, Corning). Dilution series of 0%, 25%, 50%, 75% and 100% were prepared with vehicle medium (VC or 0%). ICP-OES was used to analyse the release of soluble calcium, phosphate, and silica ions in the extracts. XRD was used to evaluate the deposition of hydroxyapatite on the samples after incubation. Prior to treating cells, all conditioned media were reconstituted with 10% (v/v) Fetal Bovine Serum (FBS), 1% (v/v) Penicillin/Streptomycin (P/S) and 1% (v/v) L-glutamine.

Fresh unprocessed human-bone marrow stromal cells (h-BMSCs, #PT2501, LONZA) were expanded and used up to passage 5 maximum. On the day prior to the cytotoxicity study, h-BMSCs were detached by enzymatic digestion (trypsin/EDTA) and seeded to 1 × 10^4^ cells well^-1^ in the central wells of 96-well plate prefilled with 60 μL of freshly prepared non-selective growth medium. The cells were incubated for 24 h in standard sterile culture conditions (37°C, 5% CO_2_) to allow the formation of a semi-confluent monolayer. Just prior to starting the *in vitro* cytotoxicity study, growth medium was replaced by freshly prepared conditioned media or VC.

Cell viability was assessed using an Alamar Blue HS kit, as per manufacturer’s protocol. A decrease of Alamar Blue dye fluorescence reading in h-BMSCs exposed to hybrids extracts was used as marker of cytotoxicity. Cytotoxicity in h-BMSCs was recorded prior to and after 24 h of exposure to the dilution extracts from conditioned media.

## Results and discussion

### Ink formulation for Direct Ink Writing

The effect of calcium content on hybrid printability, chemical characteristics, and mechanical properties were analysed, using TEOS:CME molar ratios of 60:40, 70:30, 80:20, 90:10 and 95:5. To keep the hybrid I:O ratios consistent (approx. 25:75 wt.%), the TEOS:PCL-diCOOH ratios were altered as the TEOS:CME ratio changed. The ratios and composition of each ink are summarised in Table 1, with the I:O ratio determined by TGA. The hybrids are identified by their TEOS:CME ratios, with a hybrid with a molar ratio of x:y being named “xSyC_CME_-CL”.

**FIGURE 2:**
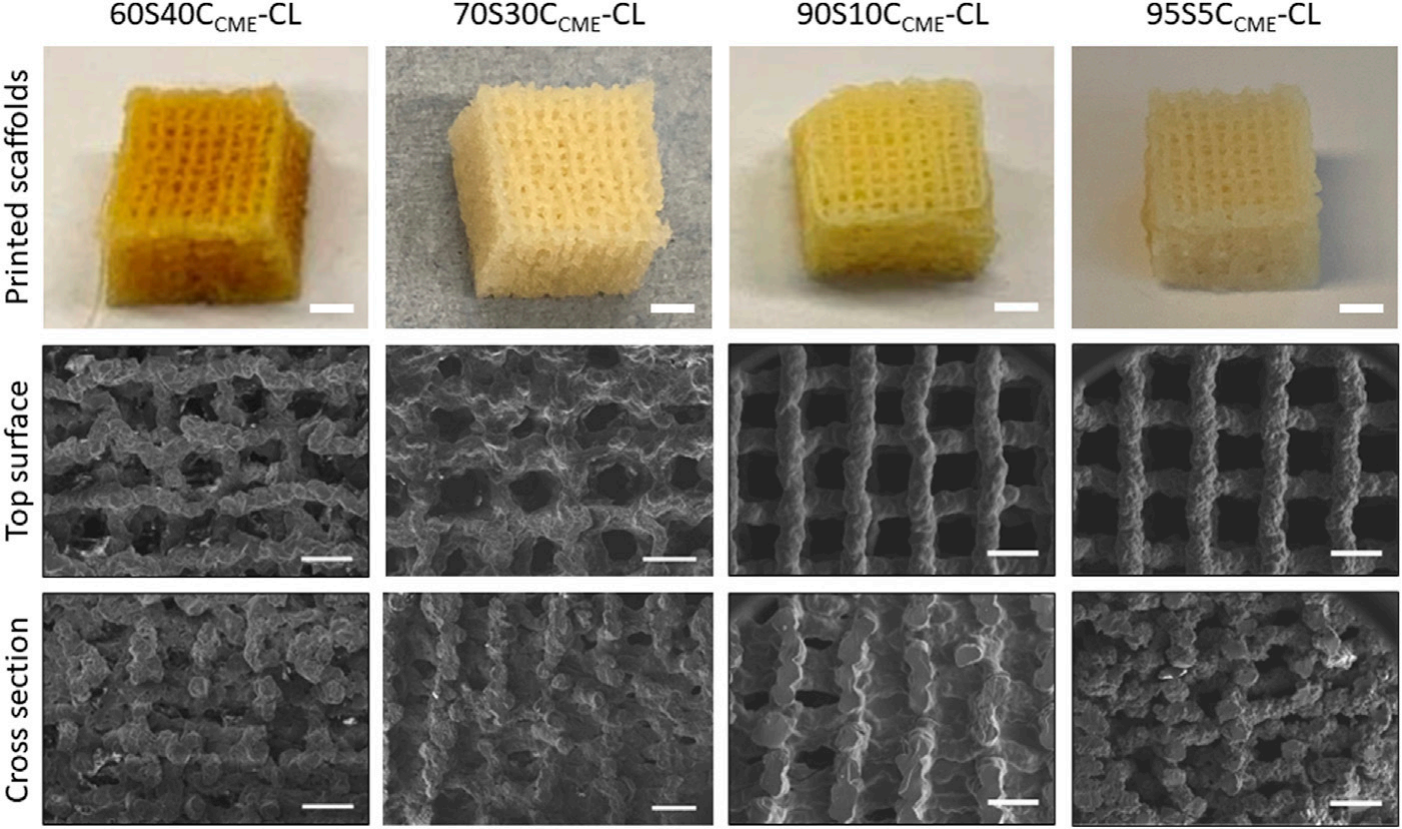
Photographs (scale bars 2 mm) and SEM micrographs (scale bars 500 µm) of 3D printed SiO_2_-CaO_CME_/PTHF/PCL-diCOOH hybrid scaffolds of compositions xSyC_CME_-CL, where x:y is the TEOS:CME molar ratio, printed via Direct Ink Writing using a tip size of 0.20 mm and strut spacing of 1 mm.

The 80S20C_CME_-CL hybrid composition was not printable due to phase separation. This composition was therefore used in understanding the chemical bonding of calcium in the hybrid but not to assess mechanical properties, as no scaffolds could be printed. All other composition “inks” appeared homogeneous and were printable, forming regular hybrid structures with open channel interconnectivity (Figure 2). After printing, the scaffolds were aged and dried, which caused shrinkage. The aim was to achieve x-y channels of approximately 500 µm and z channels of 100 µm post drying (Table 2) by setting a strut spacing of 1 mm and using a tip size of 0.20 mm. The channels of each scaffold were measured using ImageJ from SEM micrographs (Figure 2), showing drying and shrinkage variability between the compositions. x-y channel widths ranged from 399.2 ± 95.5 to 515.2 ± 80.2 μm, shrinkage of 50%—65% compared to the CAD file. Channel widths in the *z* direction were measured at 103.2 ± 32.8 to 150.5 ± 52.5 μm, 50%—75% of the CAD file, due to shrinkage and the weight of the struts in the *z* direction. The scaffold printed from these calcium containing inks shrank more than calcium-free (100S0C-CL) hybrid scaffolds previously printed by Li et al. to achieve the same 500 μm final channel size (Li et al., 2020) (x - y channel size was 503 ± 82 μm, with strut sizes ranging between 210—230 μm^32^). This was expected due to the addition 2-methoxyethanol solvent introduced during the hybrid synthesis for the calcium addition. Increasing the amount of calcium with CME increased the gelation time to reach printability of the ink, as well as the printing window, meaning the ink was printable for longer (varying from 1 h to 2 h). It however increased the shrinkage of the hybrid scaffold during the drying stage, showing a difference of approximately 100 µm between compositions (Table 2). The aim to create scaffolds with an open interconnected channel network was achieved for hybrid compositions: 60S40C_CME_-CL, 70S30C_CME_-CL, 90S10C_CME_-CL, and 95S5C_CME_-CL. The variability observed did not impact the final structure, but the different hybrids need further testing to underline which composition shows the most potential for bone regeneration.

**TABLE 2:** Structure of SiO_2_-CaO_CME_/PTHF/PCL-diCOOH hybrids of different compositions (xSyC_CME_-CL, where x:y is the TEOS:CME molar ratio) after Direct Ink Writing using a tip size of 0.20 mm and strut spacing of 1 mm. Mean values ± standard deviation (*n* ≥ 3).

Hybrid name	x-y channels (µm)	z channels (µm)
60S40C_CME_-CL	399.2 ± 95.5	150.5 ± 52.5
70S30C_CME_-CL	473.2 ± 73.6	103.2 ± 32.8
90S10C_CME_-CL	439.8 ± 76.1	152.4 ± 71.5
95S5C_CME_-CL	515.2 ± 80.2	117.3 ± 25.3

**FIGURE 3:**
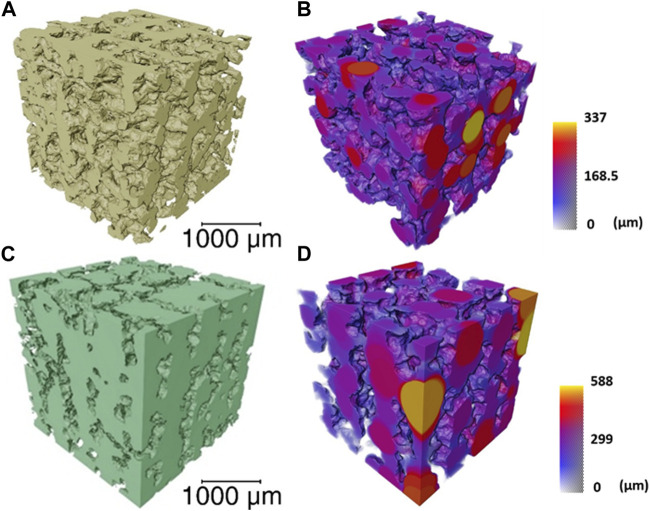
μCT imaging and analysis of a volume of interest (2,600 × 2,600 × 2,600 μm) of the 60S40C_CME_-CL hybrid scaffold of overall porosity 51.2% ± 2.8% and pore interconnectivity of 99.9%, **(A)** 3D rendering of the scaffold struts; **(B)** strut thickness map coloured by their diameter size; **(C)** 3D rendering of the interconnected pore network (negative of the strut rendering); and **(D)** a colour map to visualise the interconnecting channel thickness.

Approximately 50% porosity was achieved in the scaffolds, measure by analysis of the µCT rendering (Figure 3). This was below the porosity of trabecular bone (70%—95%) (Aspden, 2004); however, the importance of the porous structure was to have open pore channels, interconnectivity suitable for tissue ingrowth, i.e., to create a scaffold for bone to grow on rather than to recreate a bone structure directly. The 60S40C_CME_-CL hybrid scaffold had a total porosity calculated at 51.2% ± 2.8% with 99.9% of the pores were interconnected (i.e., connected to each other and the surface). The strut size is in Figure 3B by a 3D colour map; the mean strut equivalent diameter was 174 ± 65 μm, with a maximum of 473 µm. Due to the channel like structure of the porosity, it was difficult to classify the channels into pores and connecting apertures (as done in some prior studies (Atwood et al., 2004; Jones et al., 2006b; Jones et al., 2009; Yue et al., 2010)) but Figure 3C shows representation of the channels (with the scaffold removed); the mean pore equivalent diameter (most are channels, see Figure 3C) was 243 ± 105 μm, with a maximum of 476 µm. Figure 3D shows connections between channels, with the majority of the channels interconnected by passages over 200 µm in equivalent diameter. Interestingly the range of pore and interconnect sizes is similar, but the mean pore equivalent diameter is larger.

#### Mechanical properties

**FIGURE 4:**
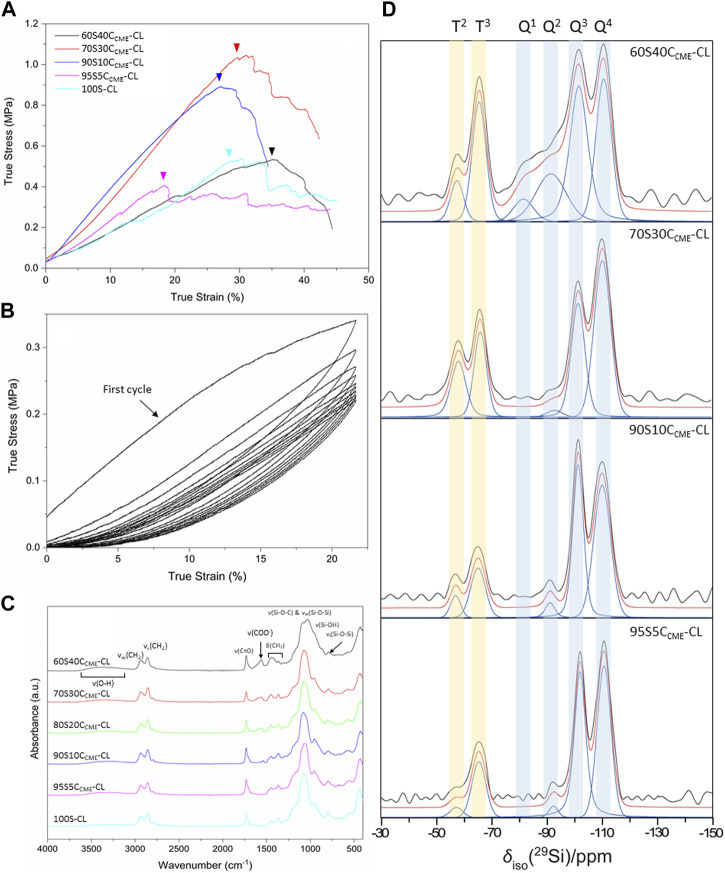
SiO_2_-CaO_CME_/PTHF/PCL-diCOOH hybrid scaffolds, with compositions xSyC_CME_-CL (x:y is the TEOS:CME molar ratio) and control 100S-CL were characterised: **(A)** true stress *versus* strain under compression; **(B)** stress *versus* strain under cyclic compression (curves shown for 60S40C_CME_-CL scaffolds); **(C)** FTIR spectra; **(D)** Single pulse solid-state ^29^Si MAS NMR spectra - shaded regions highlight peaks attributed to T^n^ and Q^n^ species.

The effect of calcium on the mechanical properties of the hybrid scaffolds was assessed by compression to failure and DMA analysis. Representative stress-strain curves in Figure 4A show elastic deformation, and the values obtained from the curves are in Table 3, with the maximum stress corresponding to strut fracture, which is equivalent to the yield stress. The corresponding strain at the yield point was taken as the maximum strain of the hybrid. An increase in strength as calcium content increased was expected, hypothesised to be due to calcium cations forming stabilising complexes with lone pairs on the ester linkages along the PCL backbone, and with any remaining unreacted carboxylate terminal groups. The carbonyl oxygen has two lone electron pairs and slight excess of negative charge to form a stabilising complex with calcium, oxygen acting as a ligand in the metal complex. It could act as a monodentate or bidentate ligand, binding through one or two donor sites (Tallia et al., 2022).

**TABLE 3:** SiO_2_-CaO_CME_/PTHF/PCL-diCOOH hybrid scaffolds with composition xSyC_CME_-CL, where x:y is the TEOS:CME molar ratio and control 100S-CL true stress, true strain, toughness modulus and elastic modulus. Mean values ± standard deviation (*n* ≥ 3).

	True stress (MPa)	True strain (%)	Toughness modulus (MPa)	Elastic modulus - 1 Hz - strain 5%–9% (MPa)
60S40C_CME_-CL	0.38 ± 0.15	44.9 ± 14.6	0.11 ± 0.02	2.14 ± 0.14
70S30C_CME_-CL	0.90 ± 0.23	29.9 ± 1.9	0.22 ± 0.04	4.48 ± 0.29
90S10C_CME_-CL	0.71 ± 0.17	30.4 ± 13.5	0.14 ± 0.04	3.26 ± 0.45
95S5C_CME_-CL	0.33 ± 0.10	24.8 ± 16.9	0.10 ± 0.06	3.54 ± 0.06
100S-CL	0.36 ± 0.14	31.3 ± 9.5	0.06 ± 0.01	-

The 95S5C_CME_-CL hybrids, which had the addition of 5 mol.% calcium compared to the 100S-CL (Ca-free) control, had a similar yield stress to the 100S-CL scaffolds, but the yield strain decreased by 6%. The slight reduction could be due to the less regular scaffold print due to the ink behaviour. As calcium content was increased, 90S10C_CME_-CL and 70S30C_CME_-CL hybrids showed an increase in yield stress by 0.3 and 0.5 MPa, respectively, to 0.71 ± 0.17 and 0.90 ± 0.23 MPa. This behaviour followed the expectation that calcium incorporation increases the hybrid strength (Tallia et al., 2022). Interestingly, the yield strain of 90S10C_CME_-CL and 70S30C_CME_-CL were similar to that of the Ca-free (100S-CL) scaffolds; however, properties of 90S10C_CME_-CL had large variation between scaffolds tested. The 70S30C_CME_-CL hybrid scaffolds exhibited the highest yield strength at 0.90 ± 0.23 MPa, at a strain of 30%, over all compositions tested, and the highest modulus of toughness (0.22 ± 0.04 MPa). As calcium content was increased further to 60S40C_CME_-CL, the yield stress dropped down to 0.38 MPa and the yield strain increased 13% compared to the control, illustrating the change in behaviour of the hybrid due to the addition of calcium. This increase in strain in the 60S40C_CME_-CL hybrid scaffold showed the shear thinning property of the CME solution affected the final properties of the hybrid. The elastic region could not be accurately quantified from measuring the Young’s modulus as it exhibited a non-linear elastic deformation. Therefore, DMA was performed to quantify the elastic modulus at 1 Hz frequency, representative of the frequency range investigated, 0.1–10 Hz and physiological activities of humans (Khusainov et al., 2013). The strain ranges of 1%–5%, 5%–9% and 9%–13% were studied as deemed to be a safe range (i.e., damage-free elastic region) to analyse the elastic properties of the hybrids of each composition. The first observation for all compositions was that the damping values obtained by DMA (i.e., tanδ) were all close to zero with E’ >> E’’. Due to the negligible damping values, it was concluded that all compositions showed an elastic behaviour in those strain ranges and the storage modulus could be directly related to the stiffness of the material. The 95S5C_CME_-CL hybrids and the 90S10C_CME_-CL showed similar values of stiffness, 3.54 ± 0.06 and 3.26 ± 0.45 MPa, respectively. However, the later composition had a larger variability, similarly to its stress strain values. As expected, from the stress-strain curves, the 60S40C_CME_-CL hybrid showed the lowest stiffness of 2.14 ± 0.14 MPa. 70S30C_CME_-CL reaching up to 4.48 ± 0.29 MPa.

Cyclic loading was performed on the 60S40C_CME_-CL hybrid scaffolds to show that even with the maximal addition of CME, 60 mol.% with respect to TEOS, the hybrid was still able to recover from deformation, similarly to the original 100S-CL hybrid (Tallia et al., 2018). A safe strain range was established with the compression data, 20% strain was inflicted on the scaffolds for 10 cycles (Figure 4B). Every cycle recovered the strain reached, after the first cycle. The maximum stress reduced with each cycle until it settled around 0.22 MPa. A hysteresis loop was observed, significative of a lag in deformation recovery, characteristic of viscoelastic behaviour (Tallia et al., 2018; Tallia et al., 2022). The first cycle showed initial higher stress reached, this behaviour seemed typical of hybrids tests in cubic form, with struts at the edge having a higher stress concentration and individually breaking (Tallia et al., 2018), whilst the bulk stayed intact. The Mullins effect could also have an impact; it is a characteristic of pure elastomer with mechanical softening occurring due to the transformation of hard domains to soft domains (Cantournet et al., 2009). This shows a decrease in stress on unloading compared to loading, characterised by the hysteresis visible in Figure 4B. Despite the first cycles showing a slightly different behaviour, the 60S40C_CME_-CL did sustain cyclic loading, making it an option for trabecular bone repair, with preconditioning potentially required before use. This slow reduction until settling at 0.22 MPa was not seen in the calcium-free 100S-CL hybrids (Tallia et al., 2018), the increased elasticity of 60S40C_CME_-CL scaffolds and higher strain reached could explain this initial compression.

#### Calcium incorporation into the silicate network

FTIR spectra show features of the organic and inorganic networks. Bands corresponding to bonds in the inorganic network are mostly found at wavenumbers below 1,200 cm^-1^, with the band between 1,100 and 1,000 cm^-1^ corresponding to the Si-O-Si asymmetric stretching and Si-O-C stretching (Figure 4C). For calcium contents less than 70S30C_CME_-CL, one prominent band was observed, with a small shoulder towards higher wavelengths. For the 60S40C_CME_-CL hybrid, two bands were observed, at 1,078 and 1,031 cm^-1^; the band at 1,078 cm^-1^ being of lower relative intensity. The band at 1,000–900 cm^-1^ corresponds to the Si-OH stretching vibration, characteristic of non-bridging oxygens (NBOs). This band was of higher relative intensity in hybrids with highest calcium content, merging in with the Si-O-Si and Si-O-C bands. Calcium’s role as a network modifier of the silicate network explains this disruption, ionically bonding with the silica network. The band at 790 cm^-1^, corresponding to the symmetric Si-O-Si stretching, was more intense in the control and lower calcium content hybrids.

The main difference between the FTIR spectra of the calcium-free control and 95S5C_CME_-CL hybrids compared to all other hybrid compositions was the carboxylate anion band, COO^−^, at 1,580 cm^-1^. This band was not present for both the control and the 95S5C_CME_-CL hybrid, and its intensity increased as the calcium content increased in SiO_2_-CaO_CME_/PTHF/PCL-diCOOH hybrids (90S10C_CME_-CL, 80S20C_CME_-CL, 70S30C_CME_-CL, and 60S40C_CME_-CL). It is possible that the calcium only remained bonded to the PCL-diCOOH if present in excess and sites for integration into the silica network were saturated. In the hybrid network, PCL-diCOOH will form covalent bonds with GPTMS or act as a chain terminator for THF polymerisation (Tallia et al., 2018); both these reactions will therefore reduce the amount of carboxyl bonds available in the hybrid network, mainly having carbonyl groups characteristic of the ester. It is hypothesised that the calcium cations could form stabilising complexes with lone pairs on the ester linkages along the PCL backbone as well as any remaining unreacted carboxylate terminal groups. The carbonyl oxygen has two lone electron pairs and slight excess of negative charge to form a stabilising complex with calcium, oxygen acting as a ligand in the metal complex. It could act as a monodentate or bidentate ligand, binding through one or two donor sites (Tallia et al., 2022). This should not affect the C=O band intensity.

The hypothesis is that calcium preferentially bonds to Si-O bonds in the silica network once hydrolysis of the TEOS occurs, and that the CME and TEOS homogenisation step does not result in any interactions between them. When the organic and inorganic solutions are mixed, no hydrolysis of the TEOS has occurred (no water present), so calcium bonds to the PCL-diCOOH via carboxyl or carbonyl interactions. Excess calcium remains unreacted until after the water and acid are added and hydrolysis of TEOS and GPTMS begins, the calcium is then scavenged from the carboxylate complex and as it is free in sol, can enter the silica network, bonding to the Si-O^-^ groups. If excess calcium is still present after, it will stay bonded to PCL-diCOOH, forming stabilising complexes with the lone pairs on the ester linkages and any unreacted carboxylate terminal groups. For the hybrid composition with no or a barely visible carboxylate band (95S5C_CME_-CL and 90S10C_CME_-CL hybrids), it can be assumed that all the calcium integrated into the inorganic network.

To confirm calcium entered the silica network, solid state ^29^Si MAS NMR of the different SiO_2_-CaO_CME_/PTHF/PCL-diCOOH hybrid compositions were obtained, comparing them to spectra from the calcium free control, 100S-CL (Tallia et al., 2022). The molecular conditions of silane groups were assessed by measuring the relative quantities of the Q^n^ (TEOS) and T^n^ (GPTMS) species (Figure 4D) and calculating the D_c_ (Table 4). Q^n^ values indicate the number (n) of bridging Si-O-Si bonds between silicon atoms, which form the silicate network, with n having a maximum of 4. The Q^n^ values (Table 4) indicate increased incorporation of calcium into the silicate network as CME precursor increased, with % Q^4^ reducing from 54% to 41% as CME was introduced at 5% (95S5C_CME_-CL). Q^3^ and Q^2^ also increased. As calcium content was increased to 90S10C_CME_-CL and 70S30C_CME_-CL, little change was seen in the Q speciation. However, for 60S40C_CME_-CL, Q^4^ reduced to 25%, and Q^2^ increased from 2% to 16% and Q^1^ units were detected (5%), indicating more calcium incorporation.

**TABLE 4:** Chemical shifts from ^29^Si CP-MAS NMR and percentage abundance from ^29^Si single-pulse MAS NMR of silicon T and Q species (from GPTMS and TEOS, respectively), and the corresponding degree of condensation (D_c_) of the silica network of the SiO_2_-CaO_CME_/PTHF/PCL-diCOOH hybrids.

	T^2^		T^3^		Q^1^		Q^2^		Q^3^		Q^4^		D_c_
Sample ID	δ_iso_	*I*	δ_iso_	*I*	δ_iso_	*I*	δ_iso_	*I*	δ_iso_	*I*	δ_iso_	*I*	
	[ppm]	[%]	[ppm]	[%]	[ppm]	[%]	[ppm]	[%]	[ppm]	[%]	[ppm]	[%]	[%]
100S-CL Tallia et al. (2022)	−58.8	3.8	−66.0	14.5	-	-	-	-	−102.5	27.9	−111.5	53.8	91.8
95S5C_CME_-CL	−57.5	2.5	−65.4	15.2	-	-	−92.4	2.1	−102.0	39.2	−110.7	41.0	88.3
90S10C_CME_-CL	−57.1	4.3	−65.2	14.2	-	-	−91.3	3.3	−101.4	35.2	−110.1	43.1	88.2
70S30C_CME_-CL	−58.5	14.4	−66.4	17.1	-	-	−93.3	1.7	−101.8	26.3	−110.6	40.5	87.8
60S40C_CME_-CL	−57.9	6.0	−65.9	18.3	−82	5.0	−91.9	15.9	−102.0	30.2	−111	24.7	78.9

The degree of condensation calculation, Equation 1 (Table 4), confirms calcium incorporation into the silicate network. The calcium-free control showed the highest degree of condensation (91.8%), this is due to the absence of calcium as a network modifier. 95S5C_CME_-CL, 90S10C_CME_-CL, and 70S30C_CME_-CL all had a similar degree of condensation, all approximately 4% less than the control. 60S40C_CME_-CL calcium showed a drop of approximately 10% compared to these compositions. In terms of D_c_ values, from the carboxylate band in the FTIR spectra (Figure 4C), it seems the additional calcium of the 70S30C_CME_-CL composition formed stabilising complexes between the lone pairs on the ester linkages and any unreacted carboxylate terminal groups. The degree of condensation of the silica network of the 60S40C_CME_-CL composition dropped a further 10%, leading to the hypothesis of the lone pairs on the ester linkages and carboxylate terminal groups being saturated, forcing the calcium to further modify the silica network. This also explained the drop in mechanical properties seen in Figure 4A for this composition (Tian et al., 1996). Previous work by Tallia et al. on the calcium-free control, showed a degree of condensation of 91.8% (Tallia et al., 2018). Their 60S40C_CME_-CL hybrids had a degree of condensation of 84.8% (Tallia et al., 2022), indicating Ca incorporation. The degree of condensation of 60S40C_CME_-CL hybrid in this work (78.9%) was lower due to the reduction in TEOS during synthesis to enable final I:O ratios of all the compositions, to match with the 100S-CL control. The degree of condensation of the silica network agreed with FTIR spectra as the Si-OH stretch at 950 cm^-1^ (Figure 4C) increased as the calcium content in the hybrid increased, significant of a lower degree of condensation, which was expected as calcium acts as a network modifier.

#### Scaffold mechanical properties during dissolution in SBF

**FIGURE 5:**
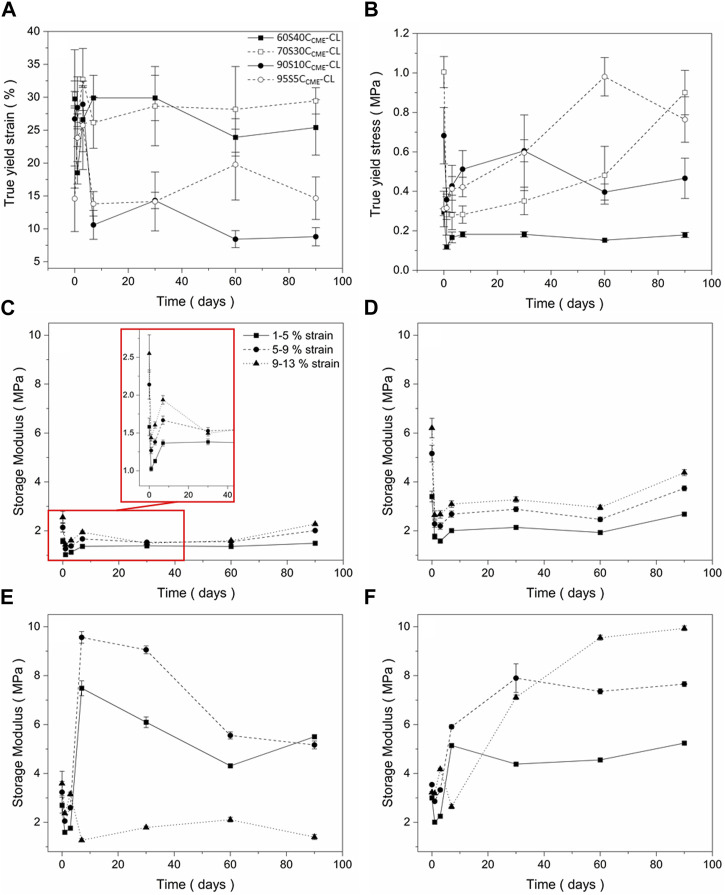
Mechanical assessment as a function of degradation, of the SiO_2_-CaO_CME_/PTHF/PCL-diCOOH hybrid scaffolds with compositions xSyC_CME_-CL, where x:y is the TEOS:CME molar ratio, from compression tests following immersion in simulated body fluid (SBF) for 0, 1, 3, 7, 30, 60, and 90 days: **(A)** true strain at yield; **(B)** true stress at yield **(C–F)** storage modulus values at strain ranges of 1%–5%, 5%–9%, 9%–13% for **(C)** 60S40C_CME_-CL, **(D)** 70S30C_CME_-CL, **(E)** 90S10C_CME_-CL, and **(F)** 95S5C_CME_-CL. Error bars are standard deviations from the mean values for *n* = 4.

The degradation properties of each composition needed to be investigated before choosing a final composition to assess osteogenesis abilities. The mechanical properties must be maintained as the scaffold decomposes *in vivo* and the degradation by-product should stimulate HCA formation of the surface of the structure, showing bioactivity. Figures 5A, B show the yield stress and strain of each hybrid scaffold composition, recorded at different times of immersion in SBF.

All compositions underwent an initial drop in yield stress after 24 h. This could be due to the change in condition, from dry to wet testing the scaffolds after immersing in SBF. The 60S40C_CME_-CL and 90S10C_CME_-CL scaffolds did not revert to their original strength and plateau at 0.2 MPa and 0.4 MPa, respectively. The 70S30C_CME_-CL and 95S5C_CME_-CL both showed a gradual increase in strength, with 70S30C_CME_-CL reaching its original value after 90 days immersion and 95S5C_CME_-CL exceeding its dry value, reaching 0.98 MPa after 60 days of immersion. The yield strain is an important factor to consider, with both 90S10C_CME_-CL and 95S5C_CME_-CL dropping to approximately 10%, indicating increase in brittleness, although 10% strain is higher than that sustained by trabecular bone. The 60S40C_CME_-CL and 70S30C_CME_-CL scaffolds showed similar strain at yield to their dry values, at approximately 30%. This showed an increased stability from these compositions, when exposed to simulated body conditions. From this behaviour, the 70S30C_CME_-CL composition was the most consistent when subjected to SBF immersion.

The storage modulus (Figures 5C–F) initially decreased over the first 3 days of immersion, for all compositions at all strain ranges. 60S40C_CME_-CL and 70S30C_CME_-CL showed little change in modulus as strain ranges increased. As immersion time increased, storage modulus reached up to 2.3 and 4.4 MPa, respectively. The 90S10C_CME_-CL scaffolds showed a sharp increase in modulus after 7 days, followed by a reduction, due to the scaffolds breaking during the test. The 95S5C_CME_-CL showed a less drastic change after 7 days, apart for the 9%–13% strain range as scaffold failure occurred during the test. This showed these two compositions were more unreliable, especially the 90S10C_CME_-CL, breaking at strains within the trabecular bone range, ≤ 7%. Values of elastic compressive modulus of trabecular bone ranges from 0.8 to 2.7 GPa (Carter and Spengler, 1978; Keaveny et al., 2004; Gibson and Ashby, 2014; Mirzaali et al., 2016; Morgan et al., 2018). The 70S30C_CME_-CL showed the highest initial modulus, whilst ensuring no failure, its value of 6.2 MPa was however not within the range required to mimic bone tissue.

#### Apatite formation in SBF

**FIGURE 6:**
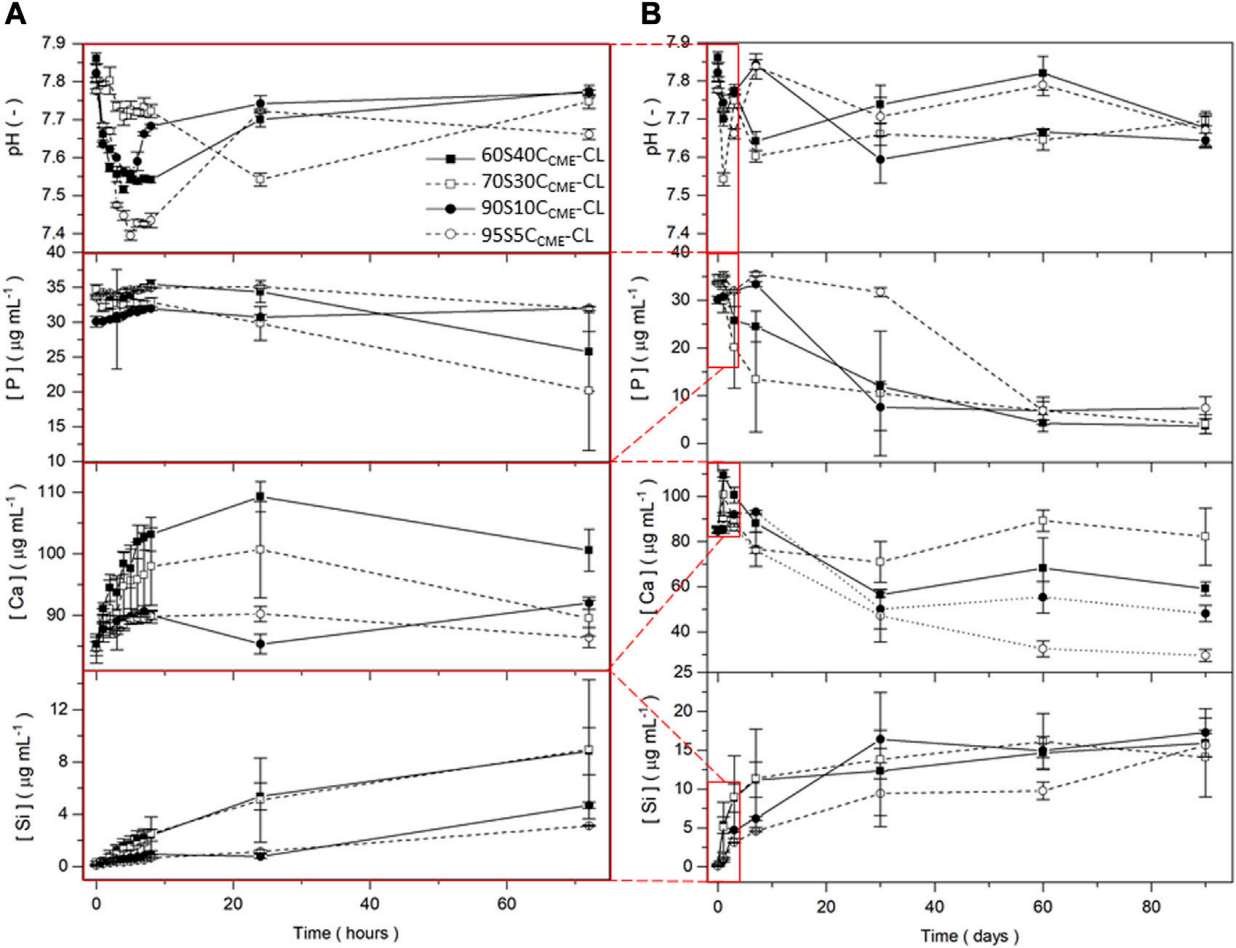
pH measurements and silicon, calcium, and phosphorous content in SBF (measured by ICP) for the SiO_2_-CaO_CME_/PTHF/PCL-diCOOH hybrid scaffolds with composition xSyC_CME_-CL, where x:y is the TEOS:CME molar ratio, for immersion up to 90 days. Expanded view of the first 72 h including hourly time points for the first 8 h are presented **(A)** to show details of ion release from the 90 days timeframe **(B)**. Error bars are standard deviations from the mean values for *n* = 5.

To evaluate the *in vitro* bioactivity of these hybrids, the pH variation in SBF was evaluated, as well as the release of silica, calcium, and phosphate ions (Figure 6). As expected, silica release was gradual from the inorganic glass network.

**FIGURE 7:**
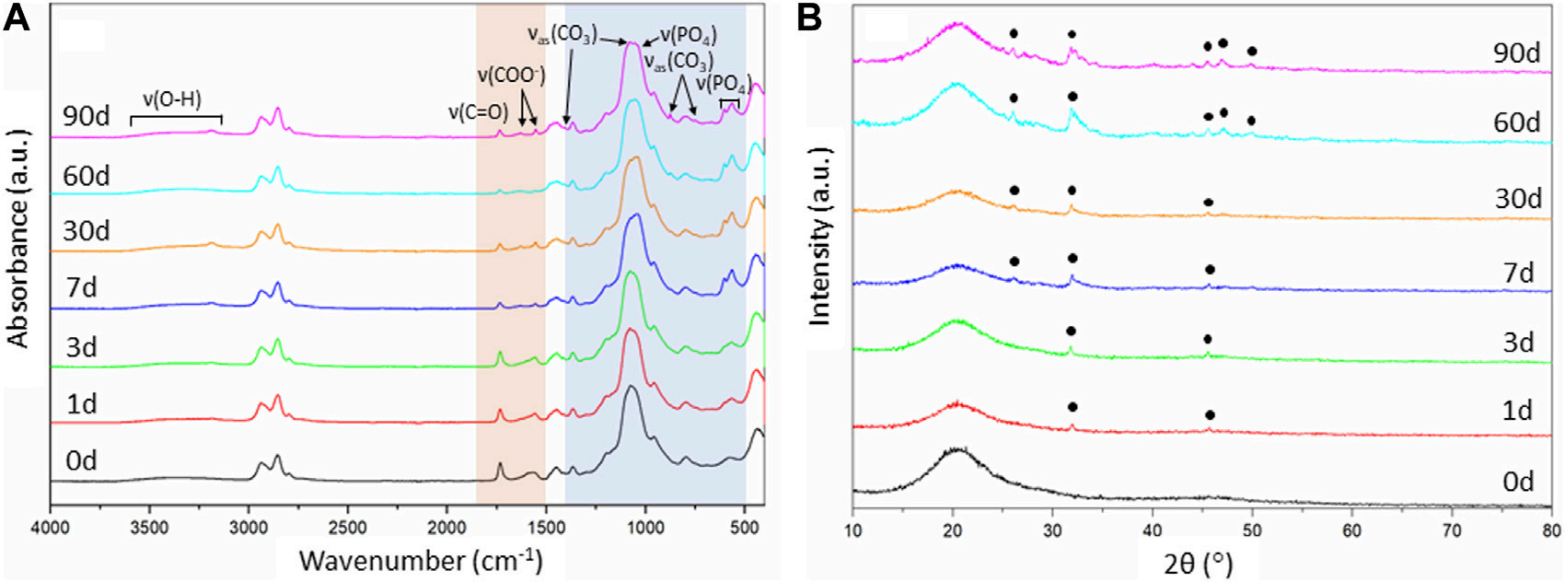
Evaluation of the SiO_2_-CaO_CME_/PTHF/PCL-diCOOH hybrid scaffolds of composition 70S30C_CME_-CL after immersion in SBF over 90 days: **(A)** FTIR spectra, section highlighted in blue is where all bands for calcium phosphate are found, the bands highlighted in orange are the carboxyl and carboxylate bands from the calcium reacting with the polymer; and **(B)** XRD patterns with annotated peaks characteristic of carbonate-hydroxyapatite (Legeros et al., 1967).

**FIGURE 8:**
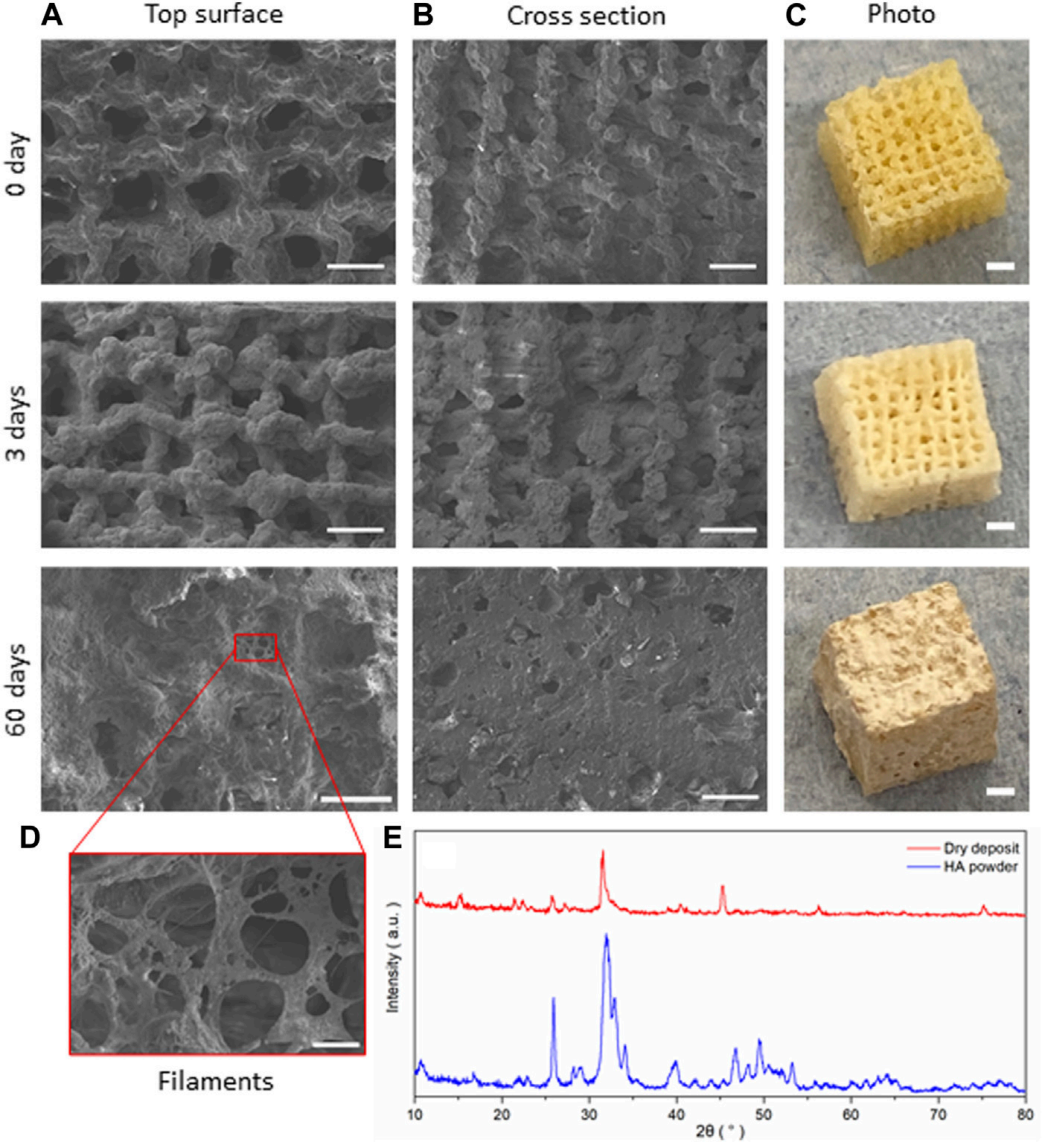
SiO_2_-CaO_CME_/PTHF/PCL-diCOOH hybrid scaffolds of composition 70S30C_CME_-CL after immersion in SBF for 0, 3, and 60 days; SEM micrographs showing **(A)** the top surface, **(B)** cross section (scale bar 500 µm), and **(C)** photographs of the scaffolds (scale bars 2 mm); **(D)** SEM image of the details of the growth on the scaffolds (scale bar 50 µm); and **(E)** the XRD patterns comparing the dried deposit from the scaffold to commercial HA powder (Sigma-Aldrich, UK).

After 7 days immersion in SBF, the 90S10C_CME_-CL and 95S5C_CME_-CL compositions released ∼5 μg mL^-1^ of Si and the 60S40C_CME_-CL and 70S30C_CME_-CL compositions released more than 11 μg mL^-1^. The calcium disruption of the silica network, seen in the NMR data Table 4, clearly increased silica network dissolution; all compositions plateaued after 30 days. Over the first 8 h, calcium released increased with calcium content in the hybrid, at rates of ∼ 1 μg mL^-1^ and 4 μg mL^-1^ per hour for the 95S5C_CME_-CL and 60S40C_CME_-CL hybrids, respectively. After 24 h, a drop in calcium concentration in the SBF was visible for all scaffold compositions, especially between day 7 and 30, from on average of 90 μg mL^-1^ down to 55 μg mL^-1^. The 70S30C_CME_-CL composition dropped to 70 μg mL^-1^, increasing back up to 90 μg mL^-1^ at day 60. Between 30 and 60 days, the calcium concentration increased for all compositions, apart from 95S5C_CME_-CL. This early behaviour was mirrored in the phosphate concentration; it was initially stable as no phosphate was released by the hybrid, and when the calcium concentration started to drop between day 1 and 3, so did the phosphate. It decreased fastest for the 70S30C_CME_-CL hybrid, followed by the 60S40C_CME_-CL, 90S10C_CME_-CL and 95S5C_CME_-CL. The drop in both calcium and phosphate after 3 days confirmed the formation of calcium phosphate, characteristic of hydroxyapatite, as seen in FTIR spectra and XRD patterns of the scaffolds after immersion (Figure 7). This correlated with the white deposit observed on the hybrids at day 3 (Figure 8). The slow increase in calcium after 30 days could be due to the rate of calcium release overtaking the rate of calcium deposition. There seems to be a limit to how much calcium phosphate can form, due to the limited phosphate content.

The pH of SBF fluctuated during the dissolution study (Figure 6), initially increasing as Ca^2+^ from the hybrid was rapidly exchanged by H^+^ from the SBF (Tallia et al., 2022) and remained between 7.4 and 7.8 for the duration of the study, which is conducive to HCA formation as OH^−^ and (CO_3_)^2-^ need to be incorporated into the calcium phosphate layer (Clark et al., 1976; Hench, 1991; Tallia et al., 2022).

As the 70S30C_CME_-CL hybrid composition was the most promising in terms of mechanical properties as a function of immersion time in SBF and showed a larger drop of calcium and phosphate in SBF, FTIR and XRD were performed to assess this formation (Figure 7).

FTIR spectra of 70S30C_CME_-CL showed the appearance of a band at around 874 cm^-1^ after 60 days in SBF (Figure 7A). This band corresponded to the formation of carbonate (Rodriguez-Blanco et al., 2011), C $O_{3}^{2-}$ . A visible shoulder at 1,409 cm^-1^ formed at the same time as the band at 874 cm^-1^, in accordance with the characteristic bands of HCA (Notingher et al., 2003). P-O bending bands (Salma et al., 2008; Malakauskaite-Petruleviciene et al., 2016) were visible after 7 days, at 550—600 cm^-1^ which are characteristic of orthophosphate, the phosphate units in HCA. P-O stretch (1,020—1,100 cm^-1^) were also present, indicative of the formation of a crystalline calcium phosphate layer (Lei et al., 2009). This layer was visible on the scaffolds after immersion in SBF (Figure 8) as a white deposit intertwined within the pores. HCA formation was confirmed using XRD. After 60 days immersion in SBF, XRD peaks were found at 26°, 32°—34°, 46°, 47° and 49° 2θ (Figure 7B), corresponding to the (002), (211), (112), (222) and (213) reflections of the hydroxyapatite phase (Balamurugan et al., 2006; Lei et al., 2009). Some of the peaks were visible as early as 1 day in SBF. The crystalline HCA peaks were slightly different to those found on bioactive glasses (Lei et al., 2009), indicating that the HCA crystals formed on the hybrid had different preferred growth orientation. These results confirm that the hybrids were able to induce HCA formation after only 1 day in SBF. Peaks increase in intensity the longer the immersion time.

Changes in the FTIR were also observed for the organic part. The carboxyl band reduced with soaking time; 1,730 cm^-1^ due to the degradation of PCL-diCOOH within the hybrid in SBF. The carboxylate band at 1,550 cm^-1^, characteristic of the excess calcium bonded to PCL-diCOOH in 70S30C_CME_-CL hybrid compositions, also reduced and almost completely disappeared, the ionic bonds breaking faster in solution, releasing the calcium at a faster rate than the covalent bonds between the PCL-diCOOH and GPTMS from the silica network or to the PTHF as a chain terminator. The carboxylate band seemed to be replaced by two very small bands at 1,550 and 1,625 cm^-1^. The calcium in the SBF could potentially initially ionically bond to PCL-diCOOH free radicals. Two bands could form from the COO^−^ interacting with different ions, such as calcium, and potentially themselves. A low intensity OH^−^ ions derived band at 630 cm^-1^ is visible for all compositions, mostly after 3 months in SBF, typical of stoichiometric hydroxyapatite (HA) (Destainville et al., 2003; Salma et al., 2008).

SEM images of the scaffolds showed little difference at low magnification after 3 days of immersion in SBF, but significant HCA formation could be seen after 7 days with the naked eye. At day 3, the scaffold showed a whiter aspect corresponding to HCA formation, which correlated with the HCA peaks being visible under XRD (Figure 7B). The deposit densified the longer the scaffold was immersed in SBF, forming filaments and plaques covering the entirety of the scaffold after 60 days (Figure 8), filling in the pores in the cross-section view. A deposit dried after 3 days immersion was investigated with XRD and compared to commercial HA powder (Sigma-Aldrich, UK), showing a close match of the peaks obtained (Figure 8E).

#### Biological response of human bone marrow stromal cells to hybrids

**FIGURE 9:**
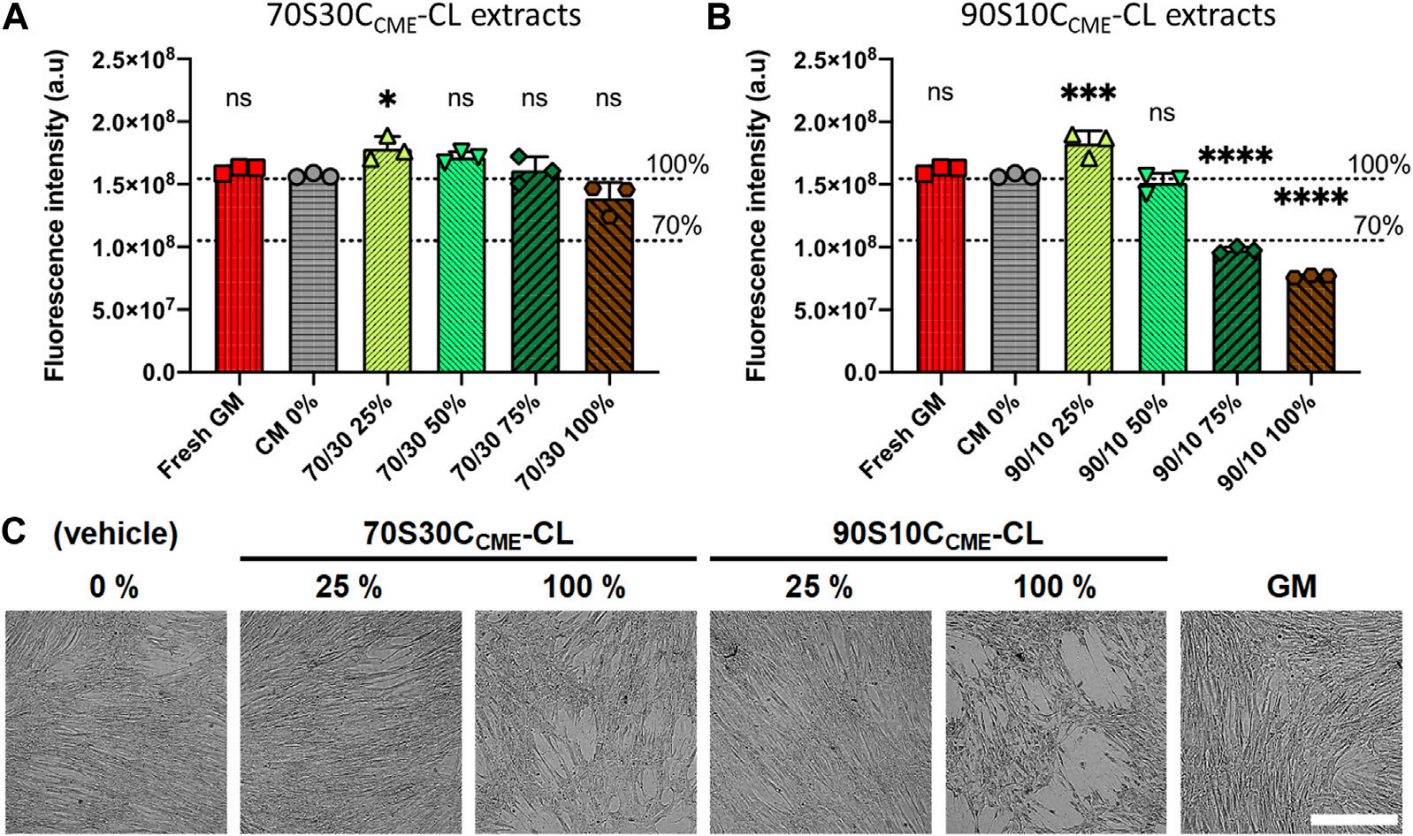
Effects of ionic release from hybrids on h-BMSC health (Alamar blue) after 24 h exposure *in vitro* (1 × 10^4^ cells well^-1^, *n* = 3). Conditioned media were prepared by soaking scaffolds in DMEM for 72 h, after which these scaffolds were removed; conditioned media were subjected to serial dilution (25, 50, 75, and 100%) for both **(A)** 70S30C_CME_-CL and **(B)** 90S10C_CME_-CL. Decrease in Alamar blue dye fluorescence intensity to below 70% of that of the CM 0% control was used as a marker of cytotoxicity. Fresh growth medium (GM) was used as an additional internal negative control for cytotoxicity. **(C)** Representative brightfield optical micrographs of h-BMSCs exposed for 24 h to hybrid extracts and controls. Scale bar 500 μm.

The performances of implantable biomaterials for bone and orthopaedic applications are determined by their interactions with cells mineralising bone tissues. *In vitro* cytotoxicity tests were performed with 70S30C_CME_-CL and 90S10C_CME_-CL conditioned media to assess the effects of ionic release on human bone marrow stromal cell (h-BMSC) health. These two hybrid compositions were chosen as they showed the highest mechanical properties and comparing two calcium contents would help understand its effect on cell health. Multipotent h-BMSCs are the most suited cells for the initial cytocompatibility assessment as they are the cells responsible for bone regeneration in bone defects. Cell health, as indicator of cytotoxic effects, was assessed in samples prior to and after 24 h exposure to serial dilution of ionic release from hybrids to basal DMEM (CM 0%) or growth medium (GM), Figure 9.

70S30C_CME_-CL hybrid ionic release did not cause cytotoxic effects in h-BMSCs, with results similar to the basal media. However, 90S10C_CME_-CL extracts did cause toxicity, unless they were diluted to at least 50%. Live-cell monitoring assessment (15 min framing rate) also confirmed loss of cell adhesion and the presence of apoptosis-like features in monolayers for 90S10C_CME_-CL extracts at 100% and 75% dilution (Figure 9C). Fluorescence readings in all 70S30C_CME_-CL conditions remained similar to levels measured in the non-exposed h-BMSCs. A slight but non-significant drop of fluorescence (equivalent to 12%) was noted in the 100% 70S30C_CME_-CL hybrid extracts. On the contrary, cell health dropped by more than 50% when cells were exposed to higher concentrations 90S10C_CME_-CL hybrid extracts.

Prior to using the extracts, ICP analysis of the media showed calcium ions in 70S30C_CME_-CL extracts were over double the concentration of that of the media conditioned with 90S10C_CME_-CL discs (Table 5). The media conditioned with the 90S10C_CME_-CL hybrid had a calcium content < 10 μg mL^-1^, less than the control media. Media conditioned with 70S30C_CME_-CL also showed a reduction in phosphate, from 32.9 ± 0.3 μg mL^-1^ before incubation to 1.2 ± 0.4 μg mL^-1^ after soaking for 72 h in vehicle. A drop of phosphate concentration associated to high levels of soluble calcium ions strongly suggests the early formation of calcium phosphate during incubation (Figure 6). Calcium is a pivotal signalling messenger to a myriad of cellular functions, from cell growth to differentiation to cell death (Viti et al., 2016). HA possesses osteoconductive properties (Khotib et al., 2021). In the 90S10C_CME_-CL hybrid composition, however, the phosphate concentration stayed equal to the vehicle baseline levels. Silica concentration in solution was minimal for the control; the 90S10C_CME_-CL composition showed a release of 26.5 ± 2.9 μg mL^-1^ and the 70S30C_CME_-CL, 70.0 ± 3.1 μg mL^-1^. Calcium acts as a network modifier and disrupts the glass network, making it more susceptible to dissolution for hybrids with higher calcium content. The release in both hybrids was higher than expected as, when incubated in SBF for 72 h, the 70S30C_CME_-CL composition had a Si concentration of ∼ 9 μg mL^-1^. The discrepancies between the *in vitro* cell and SBF ICP results are explained by different volume to weight ratios used during incubation. A typical ratio of 1.5 mg mL^-1^ hybrid disc to buffer (Jones et al., 2001) was used in the hybrid scaffold SBF studies, whereas extraction ratios followed the 3 cm^2^ mL^-1^ ISO guideline for 1 mm thick discs (equiv. 150 mg mL^-1^) in the *in vitro* cytotoxicity (ISO 10993-12:2021 Biological evaluation of medical devices—Part 12: Sample preparation and reference materials, 2021). The increased concentration explains the higher ion concentration, minimised by the fact that scaffolds show a higher surface area than discs. The higher release of calcium ions from 70S30C_CME_-CL hybrids facilitated the formation of calcium phosphate, a major component of HCA.

**TABLE 5:** Elemental concentration of phosphorous, calcium, and silicon content in vehicle medium (VC) after 72 h incubation of 70S30C_CME_-CL or 90S10C_CME_-CL hybrid discs (37°C, 5% CO_2_) (*n* = 3).

	[P] (µg mL^-1^)	[Ca] (µg mL^-1^)	[Si] (µg mL^-1^)
VC (0%, vehicle)	32.9 ± 0.3	72.9 ± 1.3	1.6 ± 0.4
90S10C_CME_-CL	32.4 ± 0.6	82.3 ± 3.2	26.5 ± 2.9
70S30C_CME_-CL	1.2 ± 0.4	187.3 ± 12.5	70 ± 3.1

**FIGURE 10:**
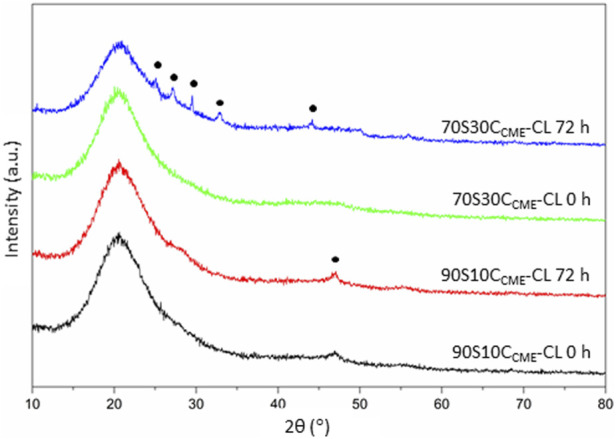
XRD patterns of the 90S10C_CME_-CL and 70S30C_CME_-CL hybrids before (0 h) and after 72 h incubation in serum-free DMEM. Annotated peaks characteristic of carbonate-hydroxyapatite (Legeros et al., 1967).

XRD analyses performed on discs after incubation confirmed the presence of phosphate and calcium in the form of HCA on the 70S30C_CME_-CL hybrids (Figure 10). The XRD patterns showed similar peak pattern than that recorded in the SBF studies on the 70S30C_CME_-CL hybrids (Figure 8). HA stimulates osteoblastic differentiation by increasing the expression of osteogenic transcription factors. However, the intracellular mechanisms involved are not fully understood yet (Khotib et al., 2021). Further studies should be focused on the 70S30C_CME_-CL hybrid composition, as it showed the best mechanical properties, HCA formation after 72 h when incubated in DMEM, and had a relative cell viability of 88% when h-BMSCs were exposed to this 100% extract serial dilution.

Further research is required to investigate how 70S30C_CME_-CL hybrid extract does not affect cell survival and growth in more complex *in vitro* models and to determine whether long-term contact to 70S30C_CME_-CL substrate is sufficient in inducing osteogenesis exists. Similarly, future work is warranted to investigate whether other key osteogenic genes are upregulated at later time points and indeed whether h-BMSCs exhibit physical characteristics of osteoblasts at these later times.

## Conclusion

SiO_2_-CaO_CME_/PTHF/PCL-diCOOH hybrids of composition 60S40C_CME_-CL, 70S30C_CME_-CL, 90S10C_CME_-CL, and 95S5C_CME_-CL were successfully synthesised, printed with pore channels of > 400 μm, and characterised. The 70S30C_CME_-CL composition reached the highest yield strength (0.90 ± 0.23 MPa), toughness modulus (0.22 ± 0.04 MPa), and storage modulus (4.48 ± 0.29 MPa). The mechanical properties and bioactivity of these hybrids were assessed in SBF up to 90 days. This confirmed the formation of HCA within all hybrid scaffolds that contained calcium in their composition. Increasing the calcium content produced faster HCA formation and strength up to a threshold of 70S30C_CME_-CL; at higher calcium content (60S40C_CME_-CL), the printability and strength of the hybrid reduced. At lower calcium contents, the hybrid was more brittle and took longer to fully form HCA, e.g., the 90S10C_CME_-CL and 95S5C_CME_-CL hybrid. The serial dilution of the 70S30C_CME_-CL extracts after dissolution in DMEM for 72 h showed a cell health not dropping below 100% for dilutions to 25, 50, and 75%. Cell health dropped by 10% relative to the non-exposed cells for the undiluted media. Apatite formation occurred on 70S30C_CME_-CL after only 72 h of incubation in DMEM. The 70S30C_CME_-CL hybrid composition also showed the best mechanical properties during degradation of 90 days, having a stable yield stress of 30% when submerged in SBF, not showing any sudden brittleness. Its yield stress was almost double that of the 60S40C_CME_-CL and 95S5C_CME_-CL compositions, which dropped when submerged, and steadily recovered over 90 days. The 70S30C_CME_-CL printed with final strut size of 100 μm and pores of 400—500 μm did not reach mechanical values close to trabecular bone. Optimisation is necessary with work ongoing using different calcium sources and altering the final implant structure and chemical composition, utilising this optimised calcium content.

## Data Availability

The raw data supporting the conclusion of this article will be made available by the authors, without undue reservation.
